# An integrated QTL-GWAS approach fine-maps a major locus (*qMWS3 − 2*) for stable resistance to maize white spot in tropical germplasm

**DOI:** 10.1186/s12870-026-08403-2

**Published:** 2026-02-24

**Authors:** Jiachen Sun, Linzhuo Li, Xingfu Yin, Fuyan Jiang, Yaqi Bi, Ranjan K. Shaw, Jiaguo Zhu, Babar Ijaz, Guohong Wang, Xingming Fan

**Affiliations:** 1 College of Agronomy and Biotechnology, Yunnan Agricultural University, Kunming, 650201 China; 2 Institute of Food Crops, Yunnan Academy of Agricultural Sciences, Kunming, 650205 China

**Keywords:** Maize white spot, GWAS, QTL, Suwan germplasm, Candidate gene, qRT-PCR

## Abstract

**Background:**

Maize white spot (MWS) is a highly destructive leaf disease and has become a major threat to global maize production in recent years. This indicates that identifying new anti-MWS genes is of vital importance. In this study, two recombinant inbred line (RIL) subpopulations derived from Suwan germplasms with MWS resistance were used.

**Results:**

By integrating quantitative trait loci (QTL) mapping and genome-wide association study (GWAS) strategies, resistance-related loci were successfully identified. The analysis results consistently indicated that there was a major resistance region on chromosome 3, where the major QTL locus *qMWS3-2*, consistently identified across three environments and BLUP values, could explain up to 10.81% of the phenotypic variation and co-localized with two significant single nucleotide polymorphisms (SNPs). Within this region, two candidate genes were identified: *Zm00001eb155730* encoding mitogen-activated protein kinase and *Zm00001eb155910* involved in sulfur metabolism. Haplotype analysis showed that the favorable haplotypes of these two genes were both associated with significantly enhanced resistance to MWS and were only present in the subpopulation derived from the highly resistant parent D39. Expression analysis further revealed a unique defense mechanism of D39. This parent strongly suppressed the candidate gene *Zm00001eb155730*, which was significantly upregulated in the highly susceptible parent. As for the defense-related gene *Zm00001eb155910*, its expression pattern is different from the induced expression of the susceptible parents Ye107 and the parent YML32.

**Conclusions:**

The candidate genes and favorable haplotypes identified in this study provide valuable resources for molecular breeding of maize and will promote the breeding process of high-resistance MWS varieties.

**Supplementary Information:**

The online version contains supplementary material available at 10.1186/s12870-026-08403-2.

## Background

Maize (*Zea mays* L.) is a major food crop, essential for global food security and economic development. In China, maize has the largest planting area and the highest output among all grains. Maize production is threatened by various diseases, including maize white spot (MWS), also known as *Phaeosphaeria* leaf spot (PLS), a serious foliar disease prevalent in maize-growing areas of tropical and subtropical regions worldwide [1–3]. First identified in India in 1965 [4], MWS has since become endemic in Brazil and the United States, causing maize yield losses of up to 60% [5, 6]. In Yunnan Province, China, MWS was first reported in 2020. As a newly emerged disease, MWS is characterized by rapid spread and extremely high infectivity. Most commercial varieties currently available are under significant threat due to their lack of resistance to MWS. By 2021, the MWS-infected area in Yunnan Province had exceeded 350,000 hectares and was rapidly spreading to neighboring provinces. Consequently, the disease is likely to become a major threat to maize production across the country [7].

The mechanisms underlying the pathogenicity of MWS are complex, and the causal pathogen appears to vary by region. Early studies identified *Phaeosphaeria maydis* as the primary pathogen [8–10]. Subsequent research suggested that additional pathogens, such as *Pantoea ananas* [6, 11, 12], and *Diaporthe eres* [13] may also contribute to the MWS. In Yunnan Province, China, where MWS has become endemic, recent investigations have revealed a diverse range of causal agents. *Epicoccum latusicollum* was identified as a major pathogen causing white spot symptoms in Kunming and Qujing regions [7]. The bacterium *Pantoea ananatis* and the fungus *Diaporthe eres* have also been confirmed as causal agents of MWS in Yunnan [13, 14]. The presence of these diverse pathogens reflects the complex etiology of MWS in Yunnan Province. It necessitates field-based resistance evaluations within this specific endemic region to identify germplasm capable of withstanding the local pathogen complex. Some researchers have proposed that environmental conditions can influence the composition of pathogens and MWS may be the result of the combined action of multiple pathogens [15]. Because of such etiological complexity, it is challenging to rely on specific control measures for a single pathogen. Breeding resistant germplasm resources remains the most effective and economical strategy for controlling MWS [3]. Therefore, the identification of resistance genes combined with marker-assisted selection can significantly accelerate the development of resistant maize varieties. The practical value of identifying resistance genes from local germplasm resources has been fully demonstrated by successful breeding cases, such as the commercial hybrid variety Yunrui 668 that exhibits MWS resistance and was developed using the parental line D39 [16].

Quantitative trait loci (QTL) mapping based on genetic linkage maps is a key approach for identifying functional genes. For instance, researchers utilized an F2:7 population derived from the B73×Mo17 to identify five QTLs, among which a QTL located at the bin7.01 region exhibited the highest *R*^2^ value of 11.0% [17]. Other researchers have detected nine QTLs related to MWS resistance using F2:3 populations, among which *qMWS8.0* has the highest *R*^2^ value of 14.8% [18]. Another study analyzed 474 test crosses and identified six QTLs related to resistance to MWS, among which a QTL located in the bin8.05 region of chromosome 8 also overlapped with a resistance locus for northern corn leaf blight [19]. However, due to limited marker resolution, most of these loci were mapped only to broad chromosomal intervals. Genome-wide association study (GWAS) is a high-resolution gene mapping method [20], and has also been increasingly used in MWS resistance research. Rossi et al. (2020) identified 9 significant single nucleotide polymorphisms (SNPs) and 13 candidate genes through GWAS using 183 tropical maize inbred lines [2]. Wang et al. (2023) identified several significant SNPs conferring resistance to MWS and determined 11 candidate genes using 143 maize inbred lines [3]. These studies have demonstrated that the genes and loci significantly associated with MWS resistance are distributed across multiple maize chromosomes and may overlap with the loci of resistance to other leaf diseases in maize. This reflects the complexity of MWS resistance as a quantitative trait.

Whole-genome resequencing (WGRS) provides high-precision and comprehensive genomic data, enabling it to identify various genetic variations including SNPs. The Suwan group represents tropical maize germplasm originating from Thailand, and it’s classified as a distinct heterotic group based on the analysis of combining ability compared to temperate groups [21]. This finding is also supported by genetic differentiation studies based on molecular markers [22]. Suwan germplasm possesses many excellent traits, such as vigorous growth, resistance to diseases, high general combining ability, and superior grain quality [21]. Some studies have reported that the Suwan germplasm has shown high levels of resistance to MWS [23] and ear rot disease [24]. Compared with temperate maize germplasm it shows higher genetic diversity and lower linkage disequilibrium (LD) [22] to serve as an ideal resource for identifying resistance loci via GWAS.

Despite some existing research foundations, the genetic basis underlying MWS resistance in the elite Suwan germplasm remains largely uncharacterized. It has rarely been used in genetic mapping studies, so its valuable resistance alleles have not been fully explored. Furthermore, previous studies often relied on traditional markers with limited resolution, which were insufficient to capture the abundant and specific genetic variations inherent in Suwan maize. To bridge these gaps, this study used two highly resistant tropical maize inbred lines (D39 and YML32, both from the Suwan group) as female parents and crossed them with the susceptible male parent Ye107 to construct mapping populations. The SNPs identified through WGRS combined with MWS resistance phenotypes were used for GWAS and QTL mapping. This study aimed to dissect the genetic basis of MWS resistance by identifying major QTLs and candidate genes via the integration of GWAS and QTL mapping. It also sought to investigate parental sequence variation and the differential expression of candidate genes, to identify elite germplasm with high MWS resistance and provide valuable genetic resources for breeding MWS-resistant maize varieties.

## Results

### Phenotypic data analysis

**Fig. 1:**
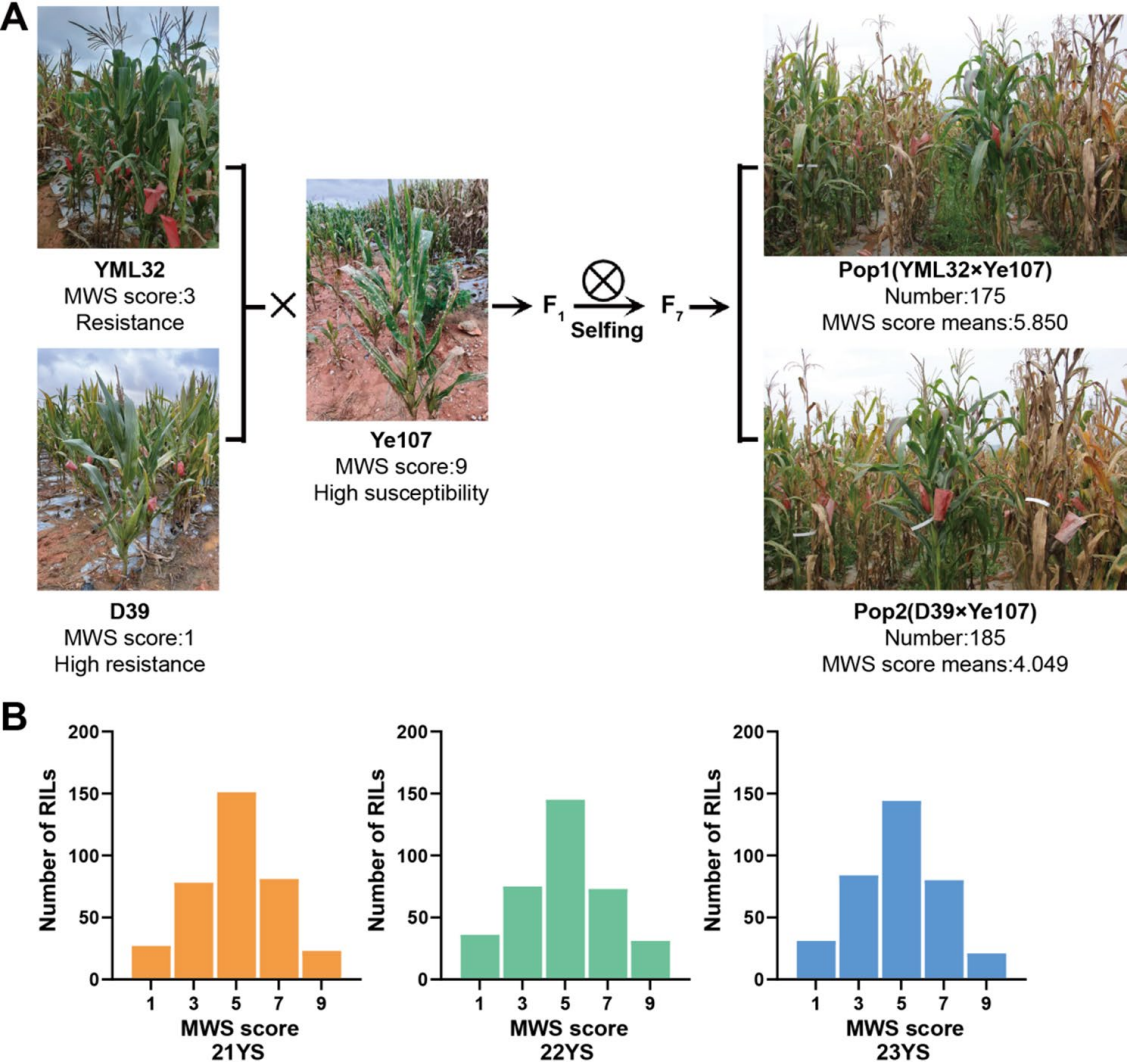
Subpopulation construction and frequency distribution of MWS score. **A** Development of the two RIL subpopulations. Ye107, serving as the common male parent of two RIL subpopulations, was used to develop 360 F_7_ RILs. **B** Frequency distribution of MWS score in 360 RILs across three environments

The incidence of MWS was investigated in three environments, 21YS, 22YS and 23YS, and MWS score for the RILs were recorded (Fig. 1A, B). The statistical analysis revealed that the average phenotypic score for the two subpopulations ranged from 3.91 to 5.93 (on a score of 1–9, where 1 indicates high resistance and 9 indicates high susceptibility), with coefficient of variation (CV) ranging from 25.8% to 52.6%. The kurtosis and skewness values across environments ranged from − 1 to 1, indicating that the MWS score of the RILs followed a normal distribution, suggesting MWS resistance is a quantitative trait. The heritability was notably high (89.7% to 94.1%), indicating that the trait was primarily controlled by genetic factors. The pairwise correlation coefficients among the subpopulations across different environments were also high (Table 1).

**Table 1:** Statistical analysis of MWS phenotypes of the two RIL subpopulations

Subpopulation	Environment	Mean	Standard Deviation	Skewness	Kurtosis	Coefficient of Variation (%)	Broad-sense heritability (%)	Correlation coefficient (%)
Pop1	21YS	5.926	1.528	0.401	-0.262	25.8	89.7	21YS/22YS = 72.4****
	22YS	5.743	1.787	0.272	-0.410	31.1		21YS/23YS = 74.9****
	23YS	5.880	1.555	0.091	0.035	26.4		22YS/23YS = 77.3****
Pop2	21YS	4.070	1.978	0.293	-0.363	48.6	94.1	21YS/22YS = 88.4****
	22YS	4.168	2.192	0.210	-0.645	52.6		21YS/23YS = 81.6****
	23YS	3.908	1.966	0.442	-0.061	50.3		22YS/23YS = 83.2****

Pearson correlation coefficients were used for the analysis

**** Indicates *P* < 0.0001

### Phylogenetic tree, principal component analysis (PCA), and population structure analysis

**Fig. 2:**
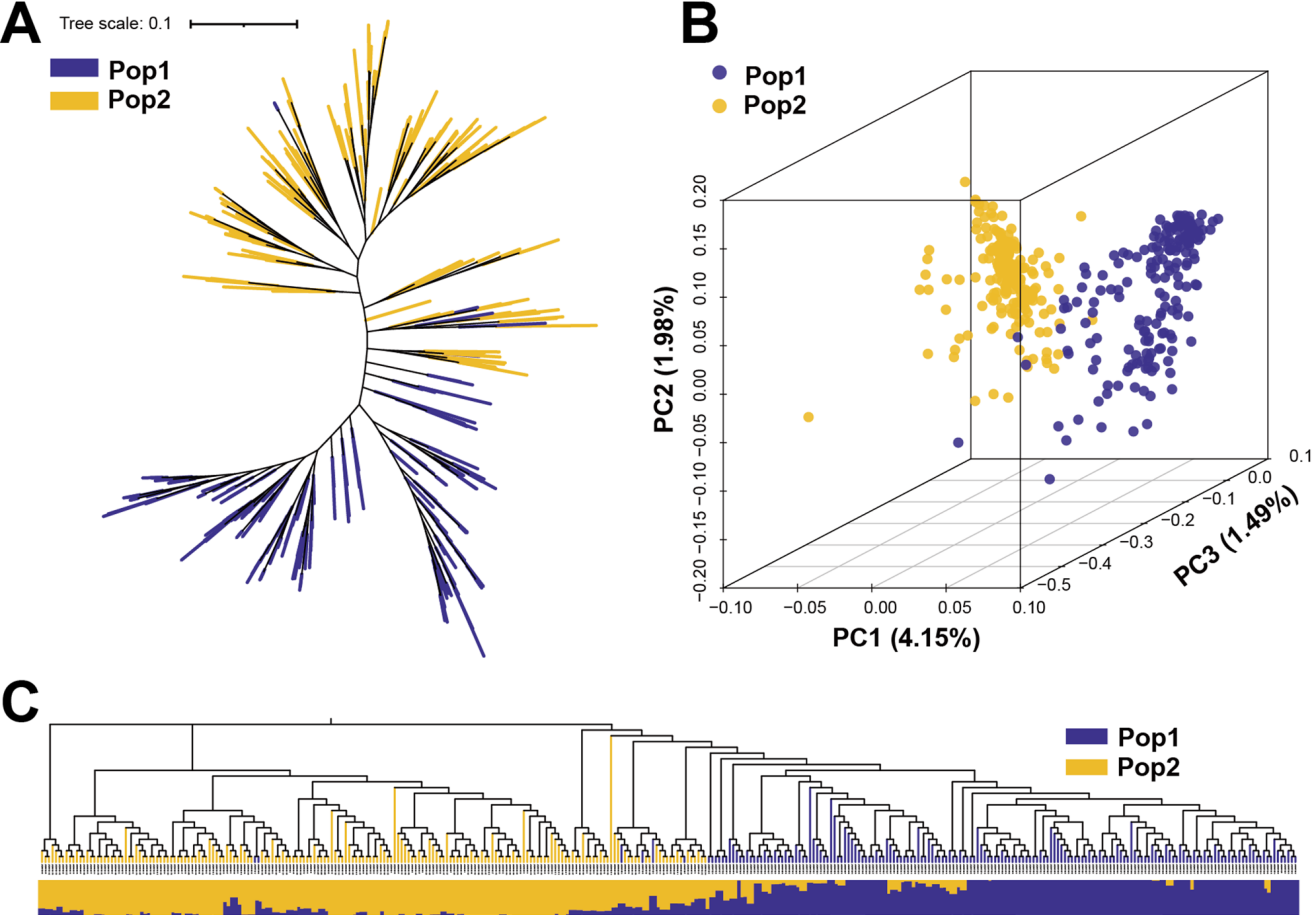
Diversity analysis of 360 RILs. **A** Phylogenetic tree. **B** Principal component analysis. **C** Population structure of the two subpopulations

The phylogenetic tree analysis revealed that the 360 maize RILs could be clustered into two major groups, with most RILs from the same subpopulation present in the same group (Fig. 2A). PCA further showed that the RILs were primarily divided into two major groups, corroborating the experimental design of this study (Fig. 2B). The population structure analysis showed that the 360 RILs were categorized into two subpopulations at K = 2 (Fig. 2C). The admixture observed between the subpopulations may have resulted from genetic drift or the use of a common male parent, Ye107, during the RIL subpopulation development. The population structure results were consistent with the findings of the PCA and phylogenetic analyses. Consequently, the first three principal components of the PCA, along with kinship matrices, were used as covariates during the GWAS analysis.

### Linkage disequilibrium (LD) decay analysis

**Fig. 3:**
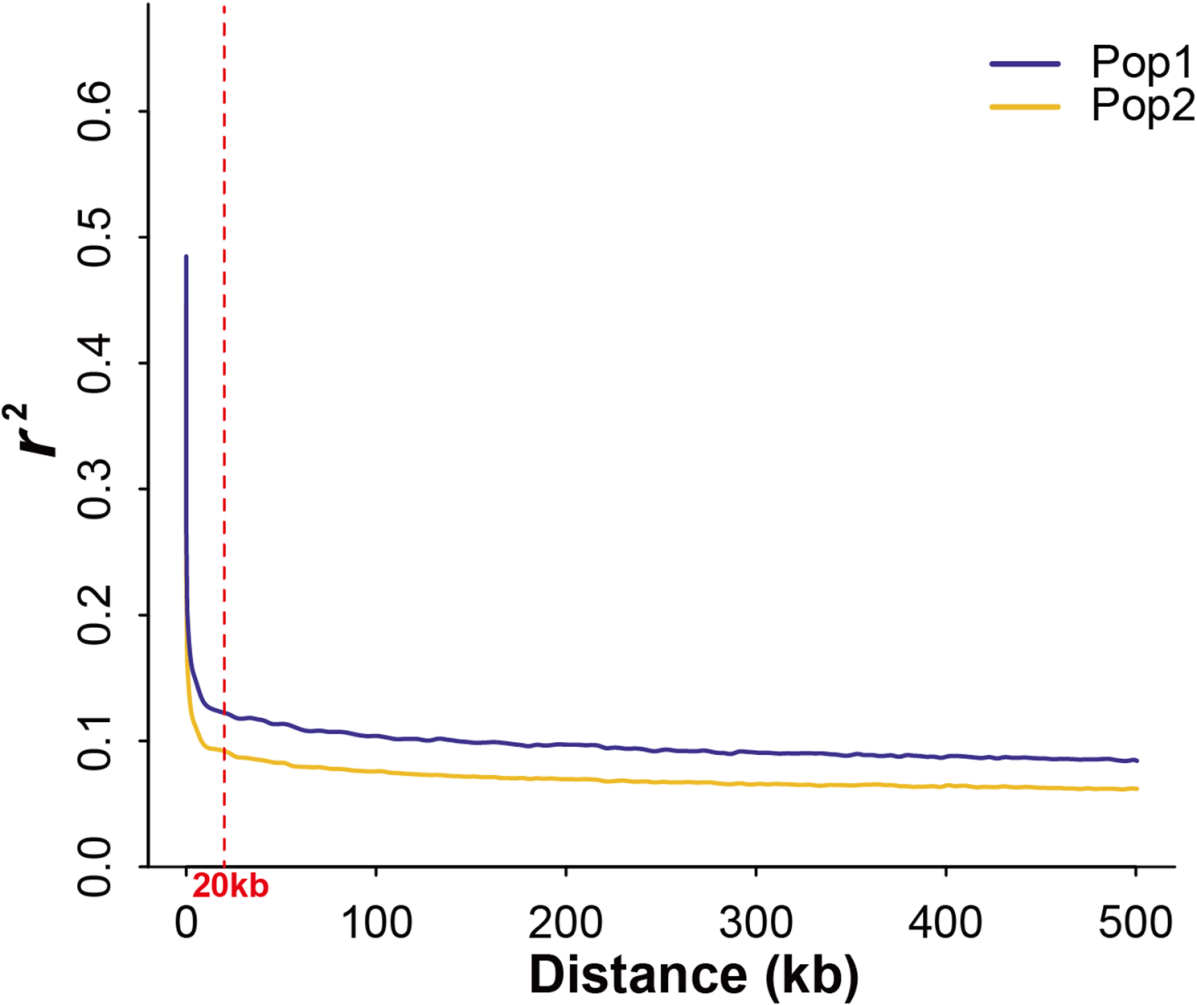
LD Decay plot of Pop1 and Pop2

Genome-wide SNPs were used to estimate the LD decay (*r*^2^) in Pop1 and Pop2. The LD decay plot (Fig. 3) revealed that LD decayed rapidly, with increasing physical distance. LD decayed to an *r*^2^ value of approximately 0.1 at a physical distance of approximately 20 kb, beyond which it gradually plateaued. Based on this LD decay distance, the candidate genes were identified by screening 20 kb regions upstream and downstream of the significant SNPs.

### GWAS of MWS resistance in maize

**Fig. 4:**
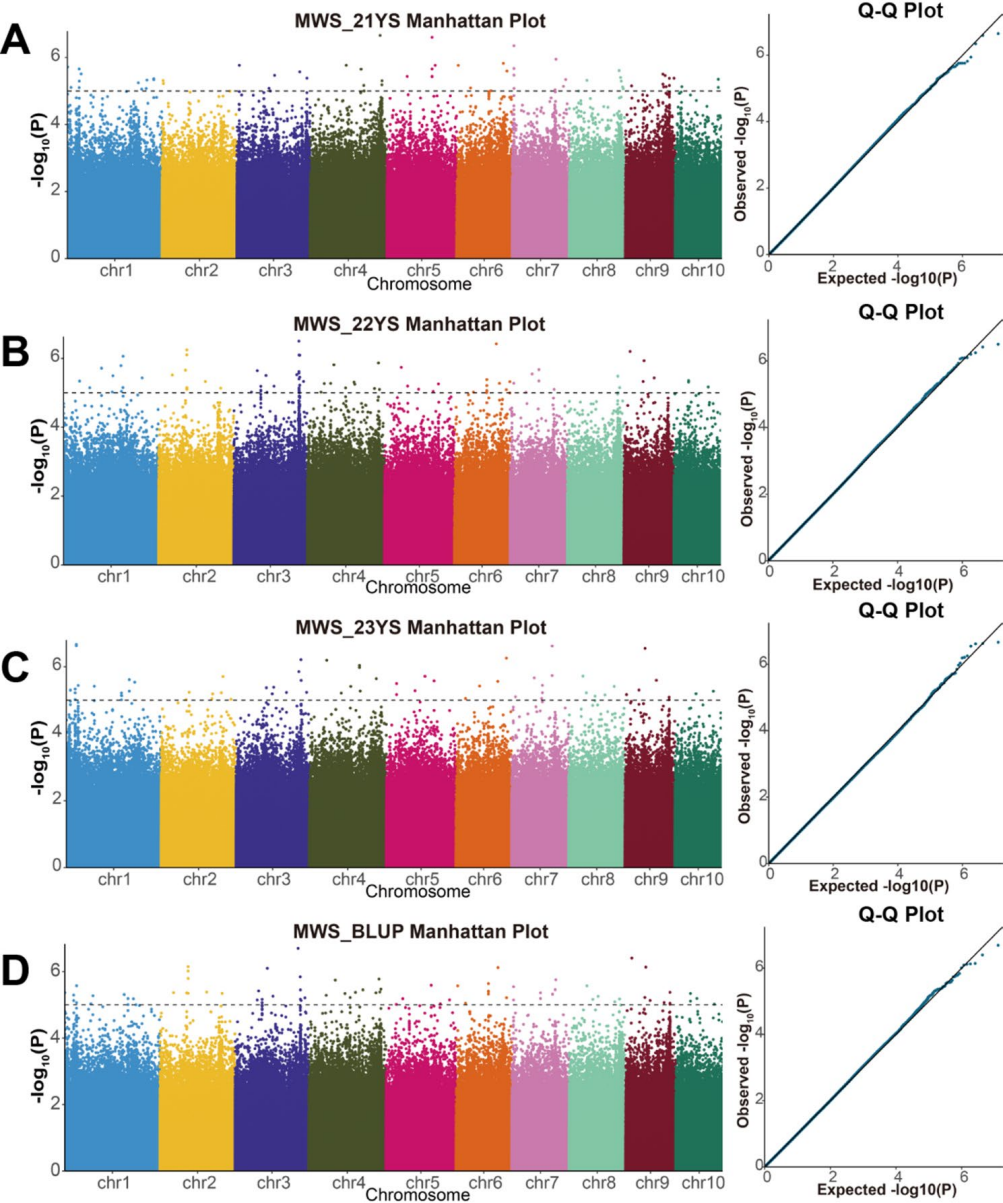
The Manhattan plots and Q-Q plots from the GWAS analysis across environments. **A** 21YS environment. **B** 22YS environment. **C** 23YS environment. **D** BLUP values

GWAS analysis of the combined set of 360 RILs revealed a total of 295 SNPs significantly associated with MWS resistance across the three environments and the BLUP values (Table S1). Among these, 63 SNPs were identified in the 21YS environment, distributed across chromosomes 1–10, with chromosome 1 containing the highest number (12) (Fig. 4A, Table S1). The phenotypic variance explained (PVE) by these SNPs ranged from 1.51% to 8.9%. In the 22YS environment, 84 significant SNPs were identified associated with MWS resistance, distributed across chromosomes 1–10, with chromosome 3 containing the most (26) significant SNPs (Fig. 4B). The PVE by these SNPs ranged from 0.33% to 11.99%. A total of 67 significant SNPs were identified as significantly associated with MWS resistance in the 23YS environment distributed across chromosomes 1–10, with chromosome 1 containing the most significant SNPs (16) (Fig. 4C). The PVE by these SNPs ranged from 1.75% to 9.75%. For the BLUP values, a total of 81 significant SNPs were identified, also distributed across chromosomes 1–10, with the most significant SNPs (12) identified on chromosomes 1 and 3 (Fig. 4D). The PVE by these SNPs ranged from 0.56% to 8.42%.

Although the number of significant SNPs varied across environments, this fluctuation reflects the quantitative nature of MWS resistance, which is known to be sensitive to environmental conditions. Variations in disease pressure across years likely influenced the detection power of minor-effect loci. Despite this dynamic, a substantial proportion of stable loci were detected. Notably, 68 SNPs were co-localized in at least two environments, indicating that the core genetic architecture remains consistent (Table S1). More strictly, 15 SNPs (Table 2) were consistently identified in at least three environments (including BLUP values). These SNPs were distributed on chromosomes 2, 3, 4, 6, 7, 8, and 9. Among these, SNP S6-164274636 exhibited the highest PVE of 9.8% in the 23YS environment. These SNPs were further used for co-localization analysis.

**Table 2:** SNPs consistently identified through GWAS in three environments

SNP	Ref	Alt	-log_10_(*P*)				Phenotypic variance explained (%)			
			21YS	22YS	23YS	BLUP	21YS	22YS	23YS	BLUP
S2-91124769	T	C	-	5.1	5.23	5.79	-	4.5	3.7	4.1
S2-201072697	G	C	-	5.13	5.7	5.34	-	5.2	3.6	4.2
S3-104127199	T	A	5.08	5.5	-	6.09	1.5	2.9	-	2.5
S3-122462798	G	A	5.46	-	5.38	5.25	7.4	-	7.3	7
S3-203999904	C	A	5.57	5.52	5.85	6.69	4.9	6.1	5.9	6.4
S3-210892574	G	T	-	6.5	6.21	5.84	-	6.9	6.5	6.3
S3-211477681	T	C	-	5.07	5.59	5.44	-	8.1	7.3	8
S4-218010298	G	A	-	5.12	5.64	5.36	-	3.3	3.2	3.4
S4-234135508	C	T	5.3	5.1	-	5.47	5.3	6.4	-	6.3
S6-136301000	A	G	-	6.42	5.56	6.11	-	6.1	4.7	4.9
S6-164274636	C	T	5.59	-	6.25	5.21	7.9	-	9.8	8.4
S7-142547432	G	A	5.94	5.1	-	5.75	5.3	6.9	-	6.5
S8-57447565	C	T	5.31	-	5.22	5.57	4	-	3.7	4.0
S9-20492780	C	T	5.15	6.2	-	6.40	5.7	4.8	-	6.1
S9-65972069	C	T	-	5.93	5.64	6.13	-	6.5	5.7	6.3

### QTL mapping of MWS resistance in maize

**Fig. 5:**
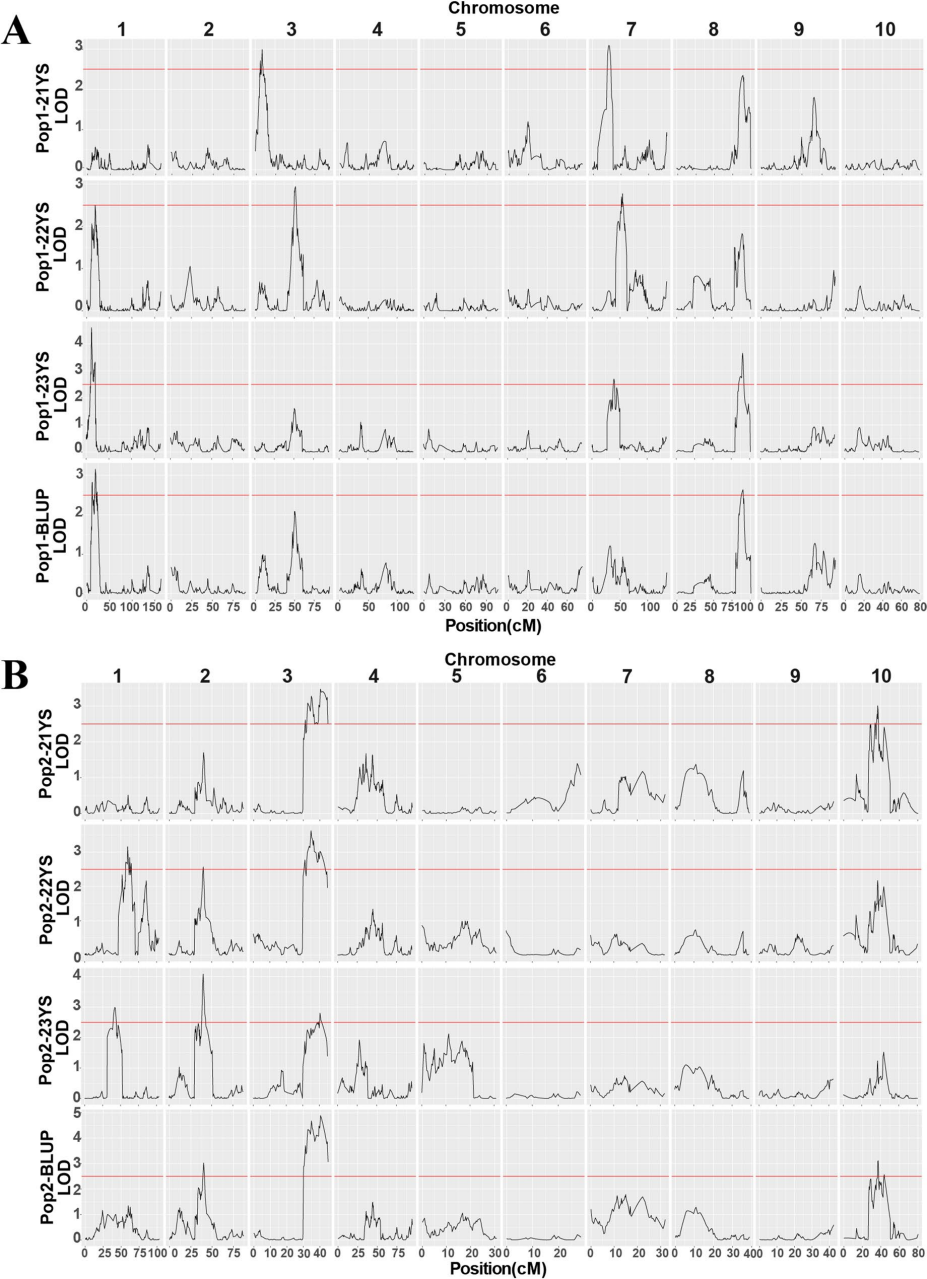
QTL mapping of MWS resistance in multiple environments. **A** QTLs identified in Pop1 for MWS resistance in the 21YS, 22YS, 23YS environments, and BLUP values. **B** QTLs identified in Pop2 for MWS resistance in the 21YS, 22YS, 23YS environments and BLUP values

During QTL mapping, a total of eight QTLs were detected on chromosomes 1, 3, 7 and 8 in Pop1 (Fig. 5A, Table 3), with LOD values ranging from 2.64 to 4.6. The PVE by these QTLs ranged from 5.45% to 9.89%. The additive effects of these QTLs ranged between − 0.47 and 0.51, with three QTLs showing positive additive effects and five showing negative effects. Similarly, in Pop2, a total of six QTLs were identified on chromosomes 1, 2, 3 and 10 (Fig. 5B), with LOD values ranging from 2.57 to 4.89. The PVE of these QTLs ranged from 5.58% to 10.81%. The additive effects varied between − 0.65 and 0.59, with one QTL showing positive effects and five showing negative effects. Notably, the LOD score of *qMWS3-2* in the BLUP dataset reached 4.89, substantially This value substantially exceeding the empirical threshold of 2.5 used in this study, demonstrating the high confidence and robustness of this major locus.

**Table 3:** Significant QTLs identified in two subpopulations across different environments

QTL	Subpopulation	Chromosome	Environment	Physical position (bp)	Genetic distance (cM)	LOD	PVE (%)	Additive effect
*qMWS1-1*	Pop2	1	22YS	141,846,260–194,375,133	57.07–64.71	3.16	6.94	-0.63
*qMWS1-2*	Pop1	1	BLUP	244,716,858–262,120,133	16.81–22.96	3.16	6.84	0.38
*qMWS1-3*	Pop2	1	23YS	250,004,342–256,744,949	39.91–43.96	2.99	6.61	-0.54
*qMWS1-4*	Pop1	1	23YS	260,537,682–283,795,885	9.68–19.86	4.6	9.89	0.51
	Pop1	1	BLUP	271,200,140–280,583,319	11.46–12.22	2.83	6.15	0.36
*qMWS2-1*	Pop2	2	BLUP	181,653,353–187,573,746	39.42–40.94	3.02	6.89	0.48
	Pop2	2	23YS	181,653,353–190,318,042	39.42–42.59	4.08	8.74	0.59
*qMWS3-1*	Pop1	3	22YS	110,926,501–114,882,104	45.95–52.48	2.94	6.36	-0.47
*qMWS3-2*	Pop2	3	BLUP	203,961,937–236,270,239	30.11–44.96	4.89	10.81	-0.62
	Pop2	3	22YS	205,780,798–225,913,893	30.83–42.9	3.63	7.88	-0.65
	Pop2	3	21YS	206,020,512–234,499,016	31.23–44.48	3.47	7.74	-0.55
	Pop2	3	23YS	218,750,741–225,913,893	39.45-45	2.8	5.89	-0.51
*qMWS3-3*	Pop1	3	21YS	210,173,589–215,907,098	6.35–9.66	2.99	6.48	-0.39
*qMWS7-1*	Pop1	7	22YS	137,209,630–140,115,325	54.69–55.7	2.77	5.97	-0.44
*qMWS7-2*	Pop1	7	23YS	153,517,386–153,830,149	39.66–40.79	2.7	5.45	-0.38
*qMWS7-3*	Pop1	7	21YS	156,121,154–159,684,276	27.26–33.29	3.09	7.44	-0.43
*qMWS8-1*	Pop1	8	BLUP	26,923,908–27,999,447	87.98–90.35	2.64	5.8	0.34
	Pop1	8	23YS	18,125,186–27,999,447	85.98–92.13	3.66	7.47	0.43
*qMWS10-1*	Pop2	10	BLUP	76,353,405–80,068,462	43.33–43.7	2.57	5.58	-0.45
*qMWS10-2*	Pop2	10	BLUP	112,438,043–119,687,237	36.49–37.53	3.11	6.72	-0.49
	Pop2	10	21YS	112,438,043–122,015,832	36.14–37.53	3.01	6.64	-0.52

Further analysis revealed two QTL regions on chromosome 3 that overlapped with several other QTL intervals. One region, spanning 210,173,589 − 215,907,098 bp, was overlapped by *qMWS3-2* (in three environments) and *qMWS3-3*. Another region, spanning 218,750,741 − 225,913,893 bp, was overlapped by QTLs *qMWS3-2* (in three environments and BLUP). Notably, *qMWS3-2* in Pop2, and *qMWS3-3* in Pop1, both exhibited negative additive effects, indicating that the resistant parent (D39 or YML32) contributed alleles associated with reduced disease severity. These two QTL regions were further explored for co-localization analysis with significant SNPs identified through GWAS.

### Co-localization analysis and candidate gene identification

**Fig. 6:**
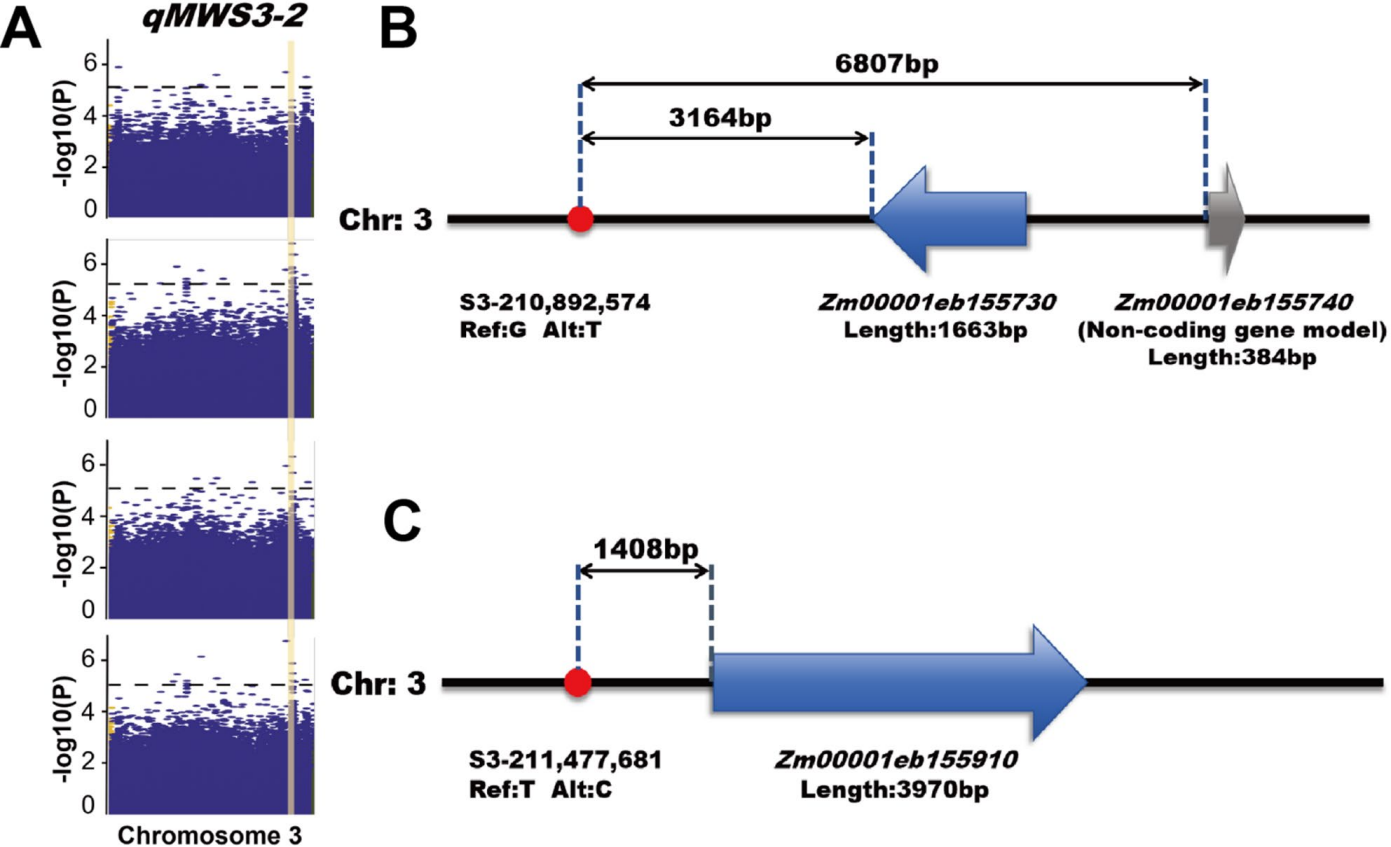
Co-localization of GWAS signals with the overlapped QTL region interval and genomic locations of candidate genes on chromosome 3. **A** Manhattan plots of chromosome 3 across three environments (21YS, 22YS, 23YS) and BLUP values. The orange vertical shaded area indicates the physical interval of the overlapped QTL region (210.17–215.91 Mb). **B** Relative positions of significant SNP S3-210892574, *Zm00001eb155730* and *Zm00001eb155740*. **C** Relative positions of significant SNP S3-211477681 and *Zm00001eb155910*

By comparing the regions of stable QTLs identified across multiple environments during QTL mapping with significant SNPs detected in multiple environments during GWAS, two SNPs, S3-210892574 and S3-211477681 were found to overlap with the regions of *qMWS3-2* and *qMWS3-3* (Table 4) (Fig. 6A). Hence, these two SNPs were selected as candidate SNPs. Candidate genes were searched within 20 kb upstream and downstream of these SNPs, consistent with the physical distance where genome-wide LD (*r*^2^) decayed to approximately 0.1 (Fig. 3). Two genes *Zm00001eb155730* and *Zm00001eb155740*, were identified 3,164 bp and 6,807 bp downstream of S3-210892574 respectively (Fig. 6B). Another gene, *Zm00001eb155910*, was identified 1408 bp downstream of S3-211477681 (Fig. 6C). Among these, *Zm00001eb155730* encodes mitogen-activated protein kinase kinase kinase 18 (MAPKKK18). *Zm00001eb155740* is a noncoding gene model, and *Zm00001eb155910* encodes D-cysteine desulfhydrase 2 in mitochondria. Given that *Zm00001eb155730* has a known functional domain relevant to stress response and is closer to the significant SNP, it was prioritized for further analysis over the more distant, non-coding gene model. *Zm00001eb155730* and *Zm00001eb155910* were selected as candidate genes for further analysis.

**Table 4:** Co-localization analysis using combined GWAS and QTL mapping

Overlapped QTL region (bp)	QTL	SNP	Gene	Functional annotation
210,173,589 − 215,907,098	*qMWS3-2* *qMWS3-3*	S3-210892574	*Zm00001eb155730*	Mitogen-activated protein kinase kinase kinase 18
			*Zm00001eb155740*	-
		S3-211477681	*Zm00001eb155910*	D-cysteine desulfhydrase 2 in mitochondria

### Haplotype analysis and variation analysis of candidate genes

**Fig. 7:**
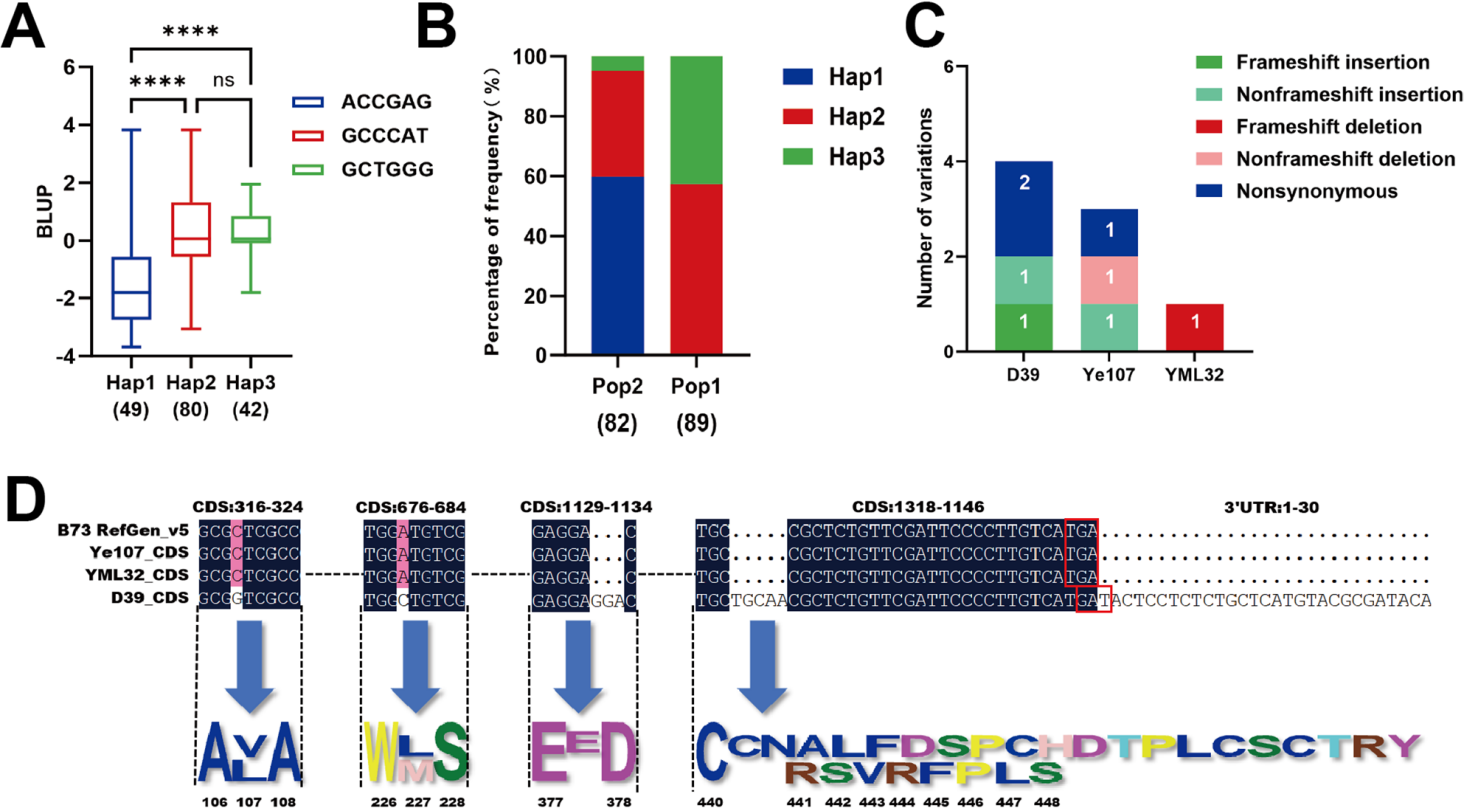
Haplotype analysis of *Zm00001eb155730* and its CDS variation in parental lines. **A** Box plot showing variation in three haplotypes of *Zm00001eb155730* for MWS resistance estimated using the BLUP values (**** Indicates *P* < 0.0001). **B** The distribution of the three haplotypes in two subpopulations. **C** Number and types of important unique homozygous variations present in the CDS region of *Zm00001eb155730* in the three parental lines. **D** Unique variation of D39 in the CDS region in *Zm00001eb155730*

Haplotype analysis of the two candidate genes was performed to study the effect of SNP variation on MWS resistance in the RILs of the two subpopulations. Three haplotypes were identified for *Zm00001eb155730*, and the distribution of Hap1, Hap2 and Hap3 in the two subpopulations was 49, 80 and 42, respectively (Fig. 7A). The BLUP values for MWS score in Hap1 were significantly lower than those of Hap2 and Hap3 (Fig. 7B), indicating that Hap1 of *Zm00001eb155730* contributes to reduced MWS score and enhanced resistance, and could be regarded as a superior haplotype. Further analysis revealed that Hap2 and Hap3 were present in Pop1 and Pop2, whereas Hap1 was only found in Pop2, suggesting that the higher MWS resistance in Pop2 was partly contributed by Hap1.

To explore the effects of important unique homozygous mutations in the gene *Zm00001eb155730* in the parental lines, a sequence comparison of its coding sequence (CDS) region among the three parental lines (D39, Ye107, and YML32) was conducted. The analysis revealed that D39, Ye107, and YML32 possessed four, three, and one notable mutations, respectively. In D39, four unique mutations were identified, including two nonsynonymous mutations, one non-frameshift insertion and one frameshift insertion (Fig. 7C). The mutation at the 318th nucleotide position caused a T→G substitution and the mutation at the 679th position caused an A→C substitution in the D39 parent (Fig. 7D). The mutation at the 318th position altered the amino acid from leucine to valine at the 107th position, causing a missense mutation. Similarly, the substitution at the 679th position altered methionine to leucine at the 227th position, also causing a missense mutation. Furthermore, the non-frameshift mutation at the 1134th position resulted in the insertion of three nucleotides (GGA), and a frameshift mutation at the 1321th position caused insertion of five nucleotides (TGCAA) in D39. The non-frameshift mutation at the 1134th position caused the addition of one glutamic acid. In contrast, the frameshift insertion at the 1321th position caused multiple amino acid alterations from positions 441 to 448, and disrupted the DNA sequence corresponding to the stop codon (TGA), extending the coding sequence into the 3’ untranslated region.

**Fig. 8:**
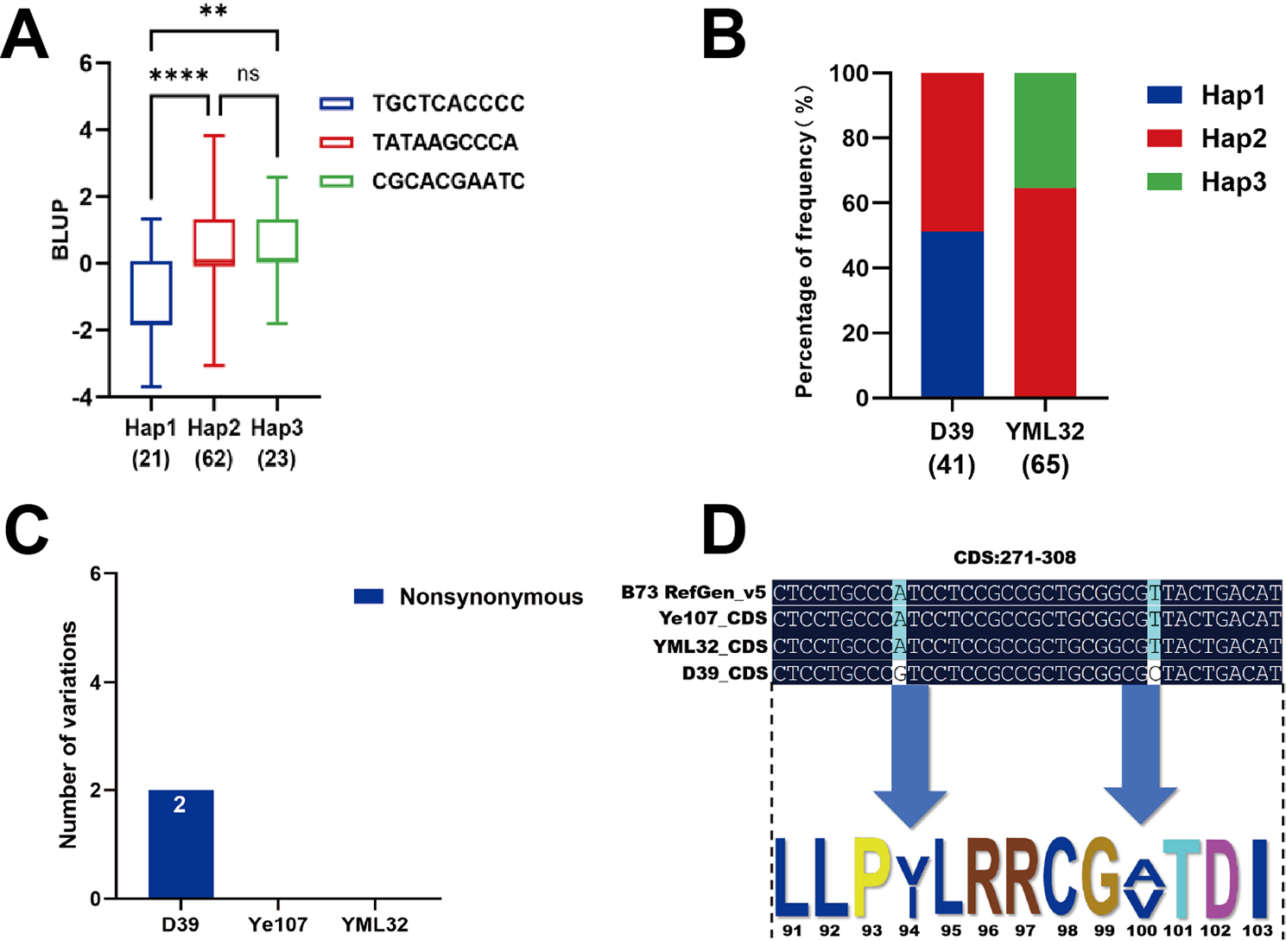
Haplotype analysis of *Zm00001eb155910* and its CDS variation in parental lines. **A** Box plot showing variation in three haplotypes of *Zm00001eb155910* for MWS resistance estimated using the BLUP values (**, **** Indicates *P* < 0.01, *P* < 0.0001). **B** Distribution of haplotypes in the two subpopulations. **C** Number and type of important unique homozygous variations present in the CDS region of *Zm00001eb155910* in the three parental lines. **D** Unique variation of D39 in the CDS region in *Zm00001eb155910*

Three haplotypes were identified for the candidate gene *Zm00001eb155910*, with the distribution of Hap1, Hap2 and Hap3 in the two subpopulations being 21, 62 and 23, respectively (Fig. 8A). The MWS score associated with Hap1 based on BLUP values was significantly lower than those of Hap2 and Hap3 (Fig. 8B), indicating that Hap1 of *Zm00001eb1555910* contributed to reduced MWS score and improved resistance, and could be considered a superior haplotype. Hap3 was identified in both Pop1 and Pop2, whereas Hap2 was found only in Pop1, and Hap1 was present only in Pop2, suggesting that the higher MWS resistance in Pop2 may be attributed to Hap1.

A sequence comparison of the CDS region of *Zm00001eb155910* among the three parental lines (D39, Ye107, and YML32) was conducted. Two variants were identified in the parental line D39, whereas no variants were detected in the CDS regions of parental lines Ye107 and YML32. In D39, two nonsynonymous mutations were identified, and both were unique to D39 (Fig. 8C). The mutation at the 280th nucleotide position caused an A→G substitution, and the mutation at the 299th nucleotide position caused a T→C substitution (Fig. 8D). The nucleotide change at position 280th resulted in a valine-to-isoleucine substitution at amino acid position 94th, causing a missense mutation. Similarly, the nucleotide change at position 299th led to a valine-to-alanine substitution at position 100th, also resulting in a missense mutation in the D39 parent. Notably, D39 was used as the female parent to develop Pop2, which exhibited higher resistance to MWS than Pop1, suggesting that the four mutations in *Zm00001eb155730* and two mutations in *Zm00001eb155910* could potentially contribute to its enhanced MWS resistance.

### qRT-PCR and expression analysis of candidate genes

**Fig. 9:**
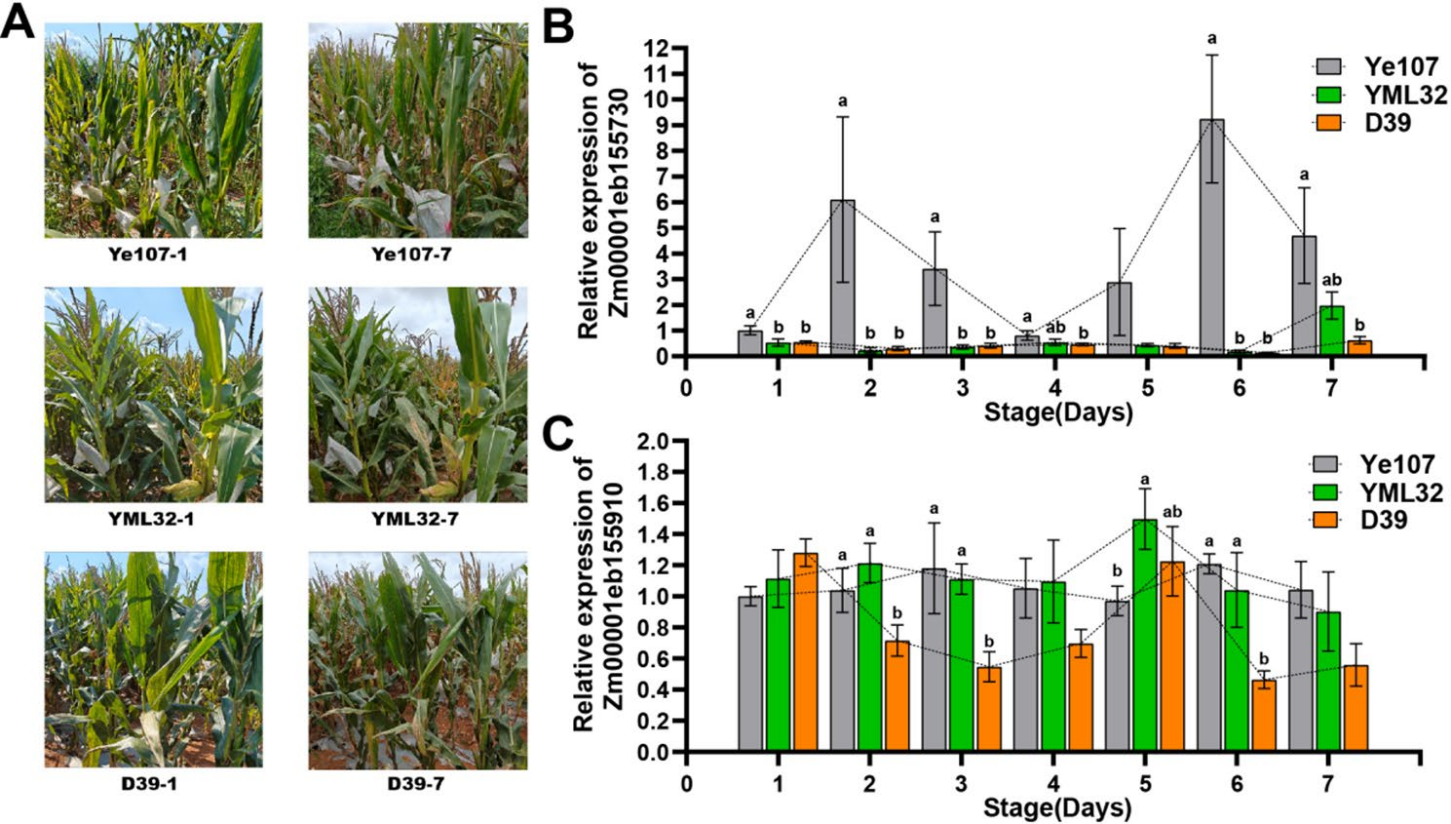
Expression analysis of candidate genes and disease progression in parental lines. **A** Phenotypic comparison of the three parental lines at stage 1 and stage 7 during natural disease development. **B** Relative transcript abundance of *Zm00001eb155730* in Ye107 (highly susceptible), YML32 (resistant), and D39 (highly resistant) over a 7-day period. **C** Relative transcript abundance of *Zm00001eb155910* in the three parental lines over the same period. Error bars represent the standard deviation (SD) of three biological replicates. Different letters above the bars indicate significant differences among the parental lines within each time point (One-way ANOVA followed by Tukey’s HSD test, *P* < 0.05)

During sampling for qRT-PCR, noticeable differences in disease progression were observed among the three parental lines. The disease spots on the leaves of Ye107 increased significantly, and the leaves had partially withered by the 7th day of sampling. In contrast, YML32 showed disease spots primarily on the lower leaves, while the leaves at the ear level and above remained relatively healthy. Remarkably, the leaves of D39 showed little to no visible change before and after sampling, and the plants were largely free of disease spots (Fig. 9A). To investigate the roles of the two candidate genes in MWS resistance, their transcript abundance was monitored over a 7-day period during rapid natural disease development in the highly susceptible parent Ye107 and the resistant parents YML32 and D39.

*Zm00001eb155730* displayed a striking inverse expression pattern between the highly susceptible and resistant lines (Fig. 9B). In the highly susceptible parent Ye107, its expression was dramatically upregulated in a two-peaks manner. Its transcript levels increased significantly at stage 2 and stage 6. In contrast, the resistant parents YML32 and D39 maintained the expression of this gene at a significantly lower level throughout the observation period. Notably, at the time points corresponding to the highest expression in Ye107, both resistant lines exhibited lowest expression levels.

*Zm00001eb155910* revealed more complex expressive dynamics among the three parents (Fig. 9C). The highly susceptible line Ye107 exhibited relatively stable and low-level expression with small variations. Conversely, the highly resistant line D39 again displayed an inverse expression trend relative to Ye107, showing significant low expression at stage 3 and stage 6, the same time points when Ye107 expression showed slight upregulation. Interestingly, the resistant line YML32 displayed a pattern where its expression upregulated (at stage 2 and stage 5) consistently one day earlier than the minor upregulation observed in Ye107.

Collectively, these distinct and dynamic expression patterns strongly suggest that both *Zm00001eb155730* and *Zm00001eb155910* are involved in the regulation of MWS resistance in maize under natural infection conditions.

## Discussion

### Maize white spot resistance of the parental lines of Suwan group

The Suwan group belonging to tropical germplasm exhibit wide genetic variation, making it an excellent resource for identifying MWS resistance genes. D39, selected from the Suwan group, is particularly known for its resistance to various maize foliar diseases, including gray leaf spot [25] and common rust [26]. In the present study, two candidate genes located on chromosome 3 were identified in the subpopulation derived from D39 across multiple environments during QTL mapping, underscoring its value as a rich genetic resource for disease resistance in maize.

Our findings confirm the high resistance of D39 to MWS, which was supported by the identification of major QTLs (*qMWS3-2*) associated with MWS resistance in Pop2. In addition, the superior haplotypes of the two candidate genes (*Zm00001eb155730* and *Zm00001eb155910*) were found only in Pop2. Important genetic variations in the CDS regions of these two candidate genes were observed in D39, which exhibited the highest frequency of these variants. (Figs. 7C and 8C). These results suggest that the resistance to MWS can be attributed to variations in D39, which contributed to the superior resistance phenotypes in the RILs of Pop2 compared to those of Pop1, derived from YML32. MWS resistance is a quantitative trait primarily governed by additive effects [27]. The negative additive effect of QTL in Pop2 derived from D39 (-0.65) was higher than that in Pop1 derived from YML32 (-0.47), indicating that the resistance in D39 was higher and heritable (Table 3).

The YML32 belonging to the Suwan group has a lower resistance to MWS than D39, but still shows resistance. The Pop1 derived from YML32 also carries a significant QTL (*qMWS3-3*), which is associated with two candidate genes (*Zm00001eb155730* and *Zm00001eb155910*). qRT-PCR analysis revealed that in YML32, *Zm00001eb155730* exhibited a suppressed expression pattern similar to that in D39. These results demonstrate the potential of Suwan germplasm as a genetic resource for MWS resistance, supported by the fact that the parental line D39 was used to develop the MWS-resistant commercial hybrid Yunrui 668 [16].

### Comparison of findings from this study with previous research

By comparing the QTLs identified in this study with previous reports, we found consistent chromosomal regions associated with MWS resistance. The QTLs identified on chromosomes 2, 3, and 10 in our study physically overlap with the intervals reported in earlier research (Table 5), including the major stable locus *qMWS3-2*. These overlapping results suggest that this genomic region may be a conserved resistance region across diverse genetic backgrounds with good stability, indicating the reliability of the loci identified in this study.

**Table 5:** Overlap of QTLs identified in previous studies with those identified in the present study

QTLs reported in the present study	Chromosome	Marker reported in previous studies	Physical location of previously identified QTLs (bp)	Reference
*qMWS2-1*	2	umc1004-umc2205	176,885,000-196,943,000	[18]
*qMWS3-2* *qMWS3-3*	3	umc1844-bnlg1257	207,033,000-218,018,000	
*qMWS3-2* *qMWS3-3*		umc1659–umc1320	200,241,761 − 213,627,968	[28]
*qMWS10-1*	10	PZE.110,041,897	80,056,274	[19]

Earlier studies by Moreira et al. (2009) and Lana et al. (2017) relied on SSR markers, which limited mapping resolution and only allowed the identification of broad chromosomal intervals [18, 28]. Lana et al. (2017) subsequently adopted a resistance gene analog (RGA) approach to identify specific genes, but this strategy prioritized candidate genes on chromosomes 4 and 8 [18]. In contrast, our study employed a combined strategy of WGRS and GWAS, successfully fine-mapping the broad overlapping interval on chromosome 3 to specific SNP loci and further identifying two candidate genes, *Zm00001eb155730* and *Zm00001eb155910*. This demonstrates the accuracy of combining WGRS-GWAS with QTL analysis.

The identification of these specific candidates reflects the uniqueness of the tropical Suwan germplasm. Unlike temperate lines used in most earlier studies, our analysis revealed that the Suwan germplasm (represented by D39) harbors unique resistance haplotypes and specific homozygous CDS variants. These superior alleles, which enrich the existing genetic resources for MWS resistance, were successfully captured in our study. Thus, although the resistance locus is spatially conserved, the discovery of these Suwan-derived variants offers new perspectives on the genetic basis of MWS resistance and provides valuable targets for diversifying maize breeding resources.

### Functional annotation of candidate genes for MWS resistance

In this study, two key candidate genes, *Zm00001eb155730* and *Zm00001eb155910*, were identified associated with MWS resistance. *Zm00001eb155730* encodes mitogen-activated protein kinase kinase kinase 18 (MAPKKK18). This is a crucial protein kinase involved in the mitogen-activated protein kinase (MAPK) signaling pathway. The MAPK cascade regulates plant immune and stress responses by converting extracellular stimuli, such as pathogen attack, into intracellular reactions [29–31]. Functional studies of MAPKKK18 orthologs in other species have provided valuable insights. For instance, *AtMAPKKK18* in *Arabidopsis* has been reported to be involved in regulating drought tolerance and abscisic acid (ABA) signaling [32, 33], while in tomato, it has been associated with bacterial wilt resistance [34]. In maize, previous research on *ZmMAPKKK18* mainly focused on abiotic stress and development, finding it associated with alkaline stress responses [35] and the regulation of internal developmental competition between fertilized grains and unfertilized ovaries [36]. Recent studies within the broader maize MAPK family have confirmed their pivotal roles in disease resistance. For example, *ZmMPK6-1* was reported to positively regulate resistance to *Exserohilum turcicum* [37], and *ZmMAPKKK45*, another Raf-like kinase, was found to confer broad-spectrum resistance to fungal pathogens by regulating ROS dynamics [38]. These findings in maize strongly support the involvement of the MAPK cascade in local biotic stress responses. The expression of *Zm00001eb155730* in two distinct peaks (Fig. 9B) is a characteristic of the host response to hemibiotrophic pathogens [39]. Although the causal agents of MWS are not yet fully elucidated, one of the confirmed pathogens, *Diaporthe eres*, belongs to the genus *Diaporthe*. This genus shares core biological characteristics with hemibiotrophic pathogens [13, 40]. Such a two-peak expression pattern is a typical manifestation of the multi-layered defense system of the plant immune system. It is often described as an initial pathogen-associated molecular pattern-triggered immunity (PTI) followed by a subsequent effector-triggered immunity (ETI) [41]. The significant differences in expression levels and the inverse temporal trends between the highly susceptible parent Ye107 and the resistant parents YML32 and D39 strongly suggest that this gene may function as a susceptibility gene or a negative regulator of immunity [42]. This hypothesis is substantiated by the functional conservation of Raf-like MAPKKKs across plant species. For instance, *AtEDR1* in *Arabidopsis* acts as a well-characterized negative regulator. Its loss-of-function mutants display enhanced resistance to powdery mildew by relieving the inhibition of defense signaling [43, 44]. Similarly, in rice, *OsEDR1* negatively regulates bacterial resistance by inhibiting the MAPK cascade [45, 46], and another Raf-like MAPKKK, *OsILA1* (*OsMAPKKK43*), has been reported to confer broad-spectrum susceptibility by suppressing the OsMAPKK4-OsMAPK6 pathway [47]. Furthermore, in cotton, the overexpression of *GhMAP3K65* has been confirmed to enhance susceptibility to pathogen infection [48]. The elevated expression of *Zm00001eb155730* in the susceptible line further suggests a potential “host-pathogen interaction” mechanism in which the pathogen exploits host signaling to facilitate infection. Excessive activation of MAPK cascades can induce programmed cell death and senescence. Given the necrotrophic phase of *Diaporthe* pathogens, premature senescence triggered by overexpression of *Zm00001eb155730* could create a nutrient-rich necrotic environment favorable for fungal colonization. Consistent with this hypothesis, overexpression of *AtMAPKKK18* in *Arabidopsis* accelerates leaf senescence through the ABA signaling pathway [49, 50]. Sequence variation analysis provides strong genetic evidence for functional inactivation of this gene in resistant parents. Unique homozygous frameshift mutations were identified specifically in the resistant parents D39 and YML32, whereas the susceptible parent Ye107 harbored only non-frameshift variants. Notably, this mutation disrupts the stop codon, and extends the coding sequence into the 3’ UTR. Such a structural alteration is likely to destabilize the protein or makes it non-functional, effectively creating a loss-of-function allele in the resistant lines. This frameshift mutation therefore represents the most biologically plausible casual variants identified in this study. We postulate that the disruption of the gene structure in the resistant lines prevents this susceptibility gene from functioning. This disruption effectively relieves the negative regulation of immunity, thereby enhancing resistance. Thus, the ability of resistant lines to suppress this gene is likely a key component of their defense mechanism, and this suppression was particularly pronounced in D39.

The D-cysteine desulfhydrase 2 (DCD2) encoded by *Zm00001eb155910* is an enzyme located in mitochondria and involved in the biosynthesis of hydrogen sulfide (H_2_S). DCD2 catalyzes the conversion of D-cysteine to H_2_S in *Arabidopsis*. It plays a vital role in various cellular processes, including stress response and plant-pathogen interactions [51]. H_2_S is established as a defense molecule against a wide range of fungal and bacterial pathogens [52]. For instance, fungal infection enhances H_2_S release in rapeseed [53]. H_2_S fumigation inhibits fungal spore germination and mycelium development in species such as *Monilinia fructicola* [54], and suppresses its pathogenicity in pear [55]. Beyond its direct antimicrobial effects, H_2_S is also closely associated with both MAPK and ABA signaling pathways [56]. Extensive research on abiotic stress has elucidated that H_2_S functions as a vital cellular regulator in the maize background. It has been demonstrated to synergize with ABA signaling to coordinate physiological responses [57], while significantly enhancing the activities of antioxidant enzymes (such as SOD, CAT, and POD) to alleviate oxidative damage [58, 59]. These studies confirm that the primary role of H₂S in the maize cellular environment is to act as a potent antioxidant buffer to prevent programmed cell death (PCD). The expression of *Zm00001eb155910* demonstrates complex expression patterns (Fig. 9C). Both the highly susceptible parent Ye107 and the resistant parent YML32 exhibited a classic inducible defense response, with YML32 showing two peaks of upregulation at stage 2 and stage 5. In contrast, the peaks in Ye107 were delayed by one stage and the amplitude of its expression change was smaller than that of YML32. This indicates that YML32’s defense response to MWS was more rapid and effective than that of Ye107. However, the highly resistant parent D39 displayed a starkly contrasting pattern of suppression relative to both Ye107 and YML32. While this suppression appears paradoxical given the defense-promoting role of H_2_S in YML32, it may instead indicate that D39 prioritizes an alternative defense pathway. H_2_S is a potent antioxidant known to scavenge reactive oxygen species (ROS) and inhibit pathogen-induced PCD [60, 61]. *Diaporthe spp*. are hemibiotrophic pathogens that require living host tissue during early infection. Sustained high H_2_S levels are known to scavenge ROS and inhibit cell death, as shown in maize abiotic stress studies [56, 57]. Such high levels could be detrimental to resistance by preventing the necessary hypersensitive response. We speculate that the suppression of *Zm00001eb155910* in D39 might function to reduce antioxidant capacity, thereby facilitating the rapid accumulation of ROS and the activation of HR to effectively restrict pathogen proliferation. To test this viewpoint, future studies will prioritize the quantification of endogenous H_2_S levels and ROS accumulation dynamics in these contrasting parents under pathogen stress.

Both genes are associated with the regulation of ABA. ABA levels are typically increased upon pathogen infection, triggering key responses such as stomatal closure, which helps conserve water and protect the plant from pathogen entry [53, 62, 63]. Although their specific roles in the context of MWS resistance require further validation, the distinct genetic variations and resistance-specific expression patterns identified in this study provide promising targets for molecular breeding.

### Future perspectives

This study successfully identified two promising candidate genes associated with MWS resistance. However, several limitations remain. The precise biological functions of these candidate genes and the broader context of host-pathogen interaction require further investigation. Future research should focus on conducting functional validation of *Zm00001eb155730* and *Zm00001eb155910*. This will help to uncover the roles of the candidate susceptibility gene and candidate hydrogen sulfide-producing defense genes in conferring resistance to MWS. To further validate the hypothesized trade-off mechanism in the highly resistant line D39, future investigations should focus on quantifying endogenous H_2_S, ROS and ABA levels, as well as measuring D-cysteine desulfhydrase enzymatic activity across different infection stages. A deeper investigation into the molecular mechanisms is required, such as identifying the interactions of the MAPKKK protein and downstream targets of the H_2_S signaling pathway. In the future, it is also necessary to clearly identify the specific causal agents of MWS in the local agricultural ecosystem, which will also provide a clearer background for the interaction between these hosts and pathogens. Nevertheless, the candidate genes and the highly resistant parent D39 identified in this study provide potential valuable genetic resources for accelerating the development of maize varieties with enhanced MWS resistance through marker-assisted breeding.

## Conclusions

This study identified two candidate genes (*Zm00001eb155730* and *Zm00001eb155910*) within the major QTL interval *qMWS-3-2* on chromosome 3. These two genes are respectively involved in the MAPK signaling pathway and sulfur metabolism, both of which play crucial roles in plant stress response and immune reaction. The differential expression patterns observed through qRT-PCR, along with the presence of superior haplotypes, further validate the functional significance of these candidate genes. The subpopulation carrying superior haplotypes as well as the sequence variations in the coding sequences of the candidate genes in the resistant parental line D39, offer valuable insights into the genetic basis of MWS resistance. These findings will contribute to the development of molecular tools for MWS resistance breeding and open new avenues for improving maize germplasm resistant to MWS. They also provide a theoretical foundation for further functional studies to elucidate the mechanisms underlying MWS resistance.

## Materials and methods

### Parental materials and field experiment design

In this study, the temperate inbred line Ye107, which is highly susceptible to MWS, was used as the male parent and crossed with two resistant inbred lines from the Suwan group, YML32 (resistant) and D39 (highly resistant), to construct two biparental RIL subpopulations. The RIL subpopulations were developed via the single seed descent (SSD) method from the F_2_ generation to the F_7_ generation (Pop1: YML32 × Ye107, *n* = 175; and Pop2: D39 × Ye107, *n* = 185), totaling 360 RILs (Table 6).

**Table 6:** Parental lines used for the construction of RIL subpopulation

Parents	Pedigree	Ecotypes	MWS score
Ye107	Derived from US hybrid DeKalb XL80	Temperate	9
YML32	Suwan 1(S)C9-S8-346-2 (Kei8902)-3-4-4-6	Tropical	3
D39	Pio. 3003-3-2-b-3-1-4-bbbb	Tropical	1

### Field experiment design and phenotypic evaluation

Field experiments of 360 RILs were conducted in Yanshan County, Yunnan Province (altitude: 1572 m, 23°36 ‘36 “N, 104°20’ 15"E) over three consecutive years (2021, 2022 and 2023). The environments were designated as 21YS, 22YS and 23YS, respectively. Yanshan County is characterized by climatic conditions that favor the annual outbreak of MWS. During the rainy season (July to August) of the experimental years, the monthly average temperature was 21.5–22.4 °C and the average relative humidity ranged from 80.6 to 86.1% (the National Aeronautics and Space Administration / Prediction of Worldwide Energy Resources platform, http://power.larc.nasa.gov), creating favorable conditions for MWS outbreaks that are consistent with previous reports [64].

Each RIL was planted in a single-row plot with a length of 4 m and a row spacing of 0.65 m, with one replication. The plant-to-plant spacing was 0.2 m and each RIL plot was evaluated 25 days after pollination for the phenotypic assessment. The average infected area of the three leaves above and below the ear was first assessed for all plants within a plot. The plot was then quantified using a 1–9 score, similar to the method of Wang et al., to obtain the disease score for each RIL [2, 3] (Table 7).

**Table 7:** Disease resistance classification of MWS

Disease score	Area occupied by disease spots	Resistance
1	≤ 5%	High resistance (HR)
3	6–10%	Resistance (R)
5	11–30%	Moderate resistance (MR)
7	31–50%	Susceptibility (S)
9	≥ 50%	High susceptibility (HS)

### Phenotypic data analysis

The MWS score of the RILs, collected across three environments were statistically analyzed using R v4.3.2. Phenotypic correlations for MWS resistance between different environments were estimated using Pearson correlation coefficients. Broad-sense heritability and best linear unbiased prediction (BLUP) were calculated to assess the genetic and environmental contributions to the phenotypes.

The broad-sense heritability was calculated as follows [65]:

$$\:{H}^{2}=\frac{{\sigma\:}_{g}^{2}}{{\sigma\:}_{g}^{2}+\frac{{\sigma\:}_{\epsilon\:}^{2}}{e}}\times\:100\%$$

where $\:{{\upsigma\:}}_{\mathrm{g}}^{2}$ represents genetic variance, $\:{{\upsigma\:}}_{{\upepsilon\:}}^{2}$ is the residual variance, and $\:\mathrm{e}$ represents the number of environments.

BLUP values for MWS score of each RIL were calculated across all environments using the R package “lme4” [66] using a linear mixed model, in which both genotype and environment were treated as random effects. The formula used was as follows:

$$\:{Y}_{ij}=u+{Line}_{i}+{Loc}_{j}+{\epsilon\:}_{ij}$$

where, Y_*ij*_ represents the phenotypic value, Line_*i*_ represents the genetic effect of the *i*th genotype, and Loc_*j*_ represents the effect of the *j*th environment. ε_*ij*_ denotes the residual error. These BLUP values were subsequently used for the GWAS analysis.

### Whole-genome resequencing and SNP identification

Whole-genome resequencing (WGRS) of the three parental lines and 360 RILs was performed. Genomic DNA was extracted from young leaf tissues using the cetyltrimethylammonium bromide (CTAB) method and purified using the QIAquick PCR purification kit (Qiagen, Valencia, CA, USA). The extracted DNA was quantified using the Qubit dsDNA HS Assay Kit (Life Technologies, Grand Island, NY, USA). The DNA was then fragmented into 200–500 bp using a Covaris ultrasonic crusher (Covaris, Woburn, MA, USA). These fragments underwent end repair, A-tailing and adapter ligation, followed by purification using AMPure XP (Agencourt, Beckmann-Coulter, USA) magnetic beads. DNA inserts of approximately 350 bp were used to construct paired-end sequencing libraries using the Illumina TruSeq DNA sample preparation kit (Illumina Inc., San Diego, CA, USA), following the standard protocols. After library construction, fragment size was evaluated using an Agilent 2100 bioanalyzer (Agilent Technologies, Santa Clara, CA, USA) and its concentration was quantified using Qubit 2.0 fluorometer (Invitrogen, Carlsbad, CA, USA).

Sequencing was performed on the Illumina NovaSeq 6000 system, generating 150 bp paired-end reads. The raw sequencing reads were filtered using fastp with default parameters to remove sequences and low-quality reads, with default parameters [67]. The filtered reads were aligned to the *Zea mays* B73 RefGen_v5 reference genome using Sentieon software (v2021-12-01) with parameters “bwa mem-k 32-M-R” [68]. The aligned reads were sorted and duplicates were removed using Samtools (parameter: rmdup) [69]. SNPs in the RILs and parental lines were identified using Sentieon software. The filtering criteria were set to exclude variants with fewer than four supporting reads, RMS Mapping Quality (MQ) <40 and Genotype Quality (GQ) <25. VCFtools v0.1.16 was used to filter the SNPs in the population. The parameters were set as: --geno 0.2 and --maf 0.05, to remove loci with a missing rate greater than 20% and a minimum allele frequency lower than 5%. Multi-allelic SNPs were filtered out, and only biallelic loci were retained [70]. High-quality SNPs were annotated using ANNOVAR software (v2021-07-16) [71].

### Principal component analysis (PCA), population structure and linkage disequilibrium (LD) analysis

An evolutionary tree was constructed using SNPs to analyze genetic relationships among the RILs of the two subpopulations. The SNPs were filtered using Plink v1.9 with the following parameters: window size 100, step size 50, *r*^2^ ≥ 0.2. VCF files were converted to PHYLIP format using vcf2phylip.py v2.0. The PHYLIP file was imported into MEGA v11.0 [72], and a neighbor-joining (NJ) tree was constructed with 1000 bootstrap values.

Principal component analysis (PCA) was conducted using Plink v1.9 to calculate the first three principal components, and the results were visualized in three-dimensional space using R v4.3.2.

The population structure was analyzed using Admixture v1.3.0 [73]. The K-value was set from 1 to 30, using default parameters. The optimal K value was selected based on the lowest cross-validation error. The population structure was visualized using the ggplot2 package of R v4.3.2.

LD analysis was conducted using genome-wide SNPs with default parameters in PopLDdecay v3.42 to calculate the degree of LD (*r*^2^) between SNP pairs at increasing physical distances. The LD decay curve was plotted using Plot_OnePop.pl [74]. The candidate gene search window was defined based on the physical distance at which LD decayed to *r*^*2*^ = 0.1. Although an *r*^*2*^ threshold of 0.2 is commonly used to indicate significant linkage, previous studies have reported that the background level of LD in maize is approximately *r*^*2*^ < 0.1 [75, 76]. Therefore, we adopted *r*^*2*^ = 0.1 as the background level of LD to define the physical extent of LD blocks. This approach provides a relatively broad candidate region while minimizing the risk of excluding potential candidate genes located in regions with moderate linkage.

### Genome-wide association study (GWAS) for MWS in maize

GWAS analysis was conducted using the linear mixed model (LMM) implemented in the genome-wide efficient mixed model association algorithm (GEMMA) software [77]. To control for confounding factors, population structure (Q matrix, represented by the first three principal components from PCA) was incorporated as a fixed effect, and individual kinship matrix was treated as a random effect to control for false positives. Since GWAS was performed separately for each environment, environmental factors were not included as covariates in these individual models. The LMM was computed using the following formula:

$$\:\mathrm{y}=\mathrm{X}\alpha\:+\mathrm{Z}\beta\:+\mathrm{W}\mu\:+e$$

where y is the phenotypic trait, X is the incidence matrix for the fixed effects, *α* is the estimated parameter of the fixed effect, Z is the incidence matrix for SNPs, *β* is the effect of SNPs, W is the incidence matrix of random effects, *µ* is the predicted random individual, and *e* is the random residual, calculated as e~(0, δe 2).

The number of independent SNPs was estimated using Plink v1.9. The significance threshold was calculated and a threshold of -log_10_(*P*) = 5 was applied to identify the SNPs significantly associated with MWS. GWAS results were visualized using Manhattan and Q-Q plots generated using CMplot v3.6.2 [78].

### Linkage map construction and QTL mapping

Based on the parental lines, the polymorphic SNPs were screened in the RILs of the respective subpopulations. The genotypic data of the RILs were filtered at threshold value of > 0.8 for data integrity and < 0.001 for segregation distortion. Subsequently, bin markers were constructed to obtain the final bin markers for each subpopulation. The bin markers were sorted using the maximum likelihood method in Joinmap4.0 [79], and genetic distances (cM) were calculated using Kosambi mapping function [80]. Finally, genetic linkage maps were constructed for the two subpopulations.

QTL mapping was conducted using composite interval mapping (CIM) in Windows QTL Cartographer v2.5 [81], configured with a walk speed of 1.0 cM and a window size of 10 cM. The method of conducting QTL mapping for each subpopulation separately was chosen to enable a clear comparison of the genetic effects contributed by the two different resistant parents. The QTLs were identified using an empirical threshold of LOD > 2.5. Although permutation tests often yield higher significance thresholds, this fixed cut-off was adopted in accordance with the established guidelines for reporting suggestive linkage in complex traits [82], allowing the detection of biologically relevant minor-effect loci. However, for the identification of major-effect QTLs, particular emphasis was placed on loci with high LOD values that significantly exceeded conventional genome-wide significance levels, ensuring the robustness of the key findings. The percentage of phenotypic variation (PVE) explained by individual QTL was calculated as the square of the partial correlation coefficient (*R*^2^). To minimize redundancy, QTLs detected within the same subpopulation across different environments (including BLUP values) that exhibited overlapping physical intervals were considered to represent the same QTL. These overlapping QTLs were merged and assigned a single unified name.

### Identification, functional annotation and haplotype analysis of candidate genes

Significant SNPs that were repeatedly detected across multiple environments and QTL regions that overlapped among these environments were separately identified. Then the SNPs and QTLs with the most significant overlap were selected. Based on this, the SNPs that were physically located within these stable QTL intervals were designated as candidate SNPs. The search window for candidate genes was defined based on the genome-wide LD decay of the subpopulation. Specifically, a 20 kb flanking region (upstream and downstream of the significant SNPs) was used for screening, as the LD (*r*^2^) decayed to the baseline level of approximately 0.1 at this distance. Functional annotation of the candidate genes was conducted using the MaizeGDB (http://www.maizegdb.org) and NCBI (http://www.ncbi.nlm.nih.gov) databases.

All SNPs within the gene bodies were extracted to construct haplotypes using TASSEL 5.0 [83]. Samples containing missing data or heterozygous loci were excluded. The phenotypic distributions of the different haplotypes were visualized using box plots generated with the ggplot2 package in R v4.3.2. Statistical significance of the differences among haplotypes was evaluated using one-way ANOVA, followed by Tukey’s honestly significant difference (HSD) test.

### Variation analysis of candidate gene in the parental lines

Sequence comparison of the coding sequence (CDS) of the candidate genes was performed by extracting the corresponding gene sequences from the reference genome, B73_RefGen_v5. Nucleotide and amino acid variations in key candidate genes across different parents were analyzed by comparing the sequences of the three parental lines: Ye107, YML32, and D39. Important unique homozygous variations located within the CDS region were analyzed.

### qRT-PCR and candidate gene expression analysis

Sampling of the parental lines Ye107, YML32 and D39 was conducted in Yanshan County during August 2023 for expression analysis of candidate genes. Leaf samples from the ear position of each parental line were collected at seven time points during the period of peak disease onset. At each time point, three biological replicates per parental line were sampled. The sampling days were designed as day 1 through 7 for each parent (e.g., stage 1, stage 2, etc.).

qRT-PCR for two candidate genes (*Zm00001eb155730* and *Zm00001eb155910*) was performed using the Tiangen SuperReal PreMix Plus (SYBR Green) kit (Tiangen, Beijing, China). Primer 5.0 software was used for primer designing. For *Zm00001eb155730*, the forward and reverse primers were as follows: F-175:5 ‘CCAGGCAAAGTGTCCA3’ and R-175:5’AACGCAAAATCAAATAGC3’. For *Zm00001eb155910*, the primers were F-164:5 ‘CGCCACTGAATGGATGCT3’ and R-164:5 ‘CGCCTTGTTGCCGTTGG3’. The *GAPDH* gene was used as internal reference for standardization [84]. The sample from Ye107 at stage 1 (Ye107-1) was used as the calibrator sample for the 2^−ΔΔCT^ method [85].

## Supplementary Information

Supplementary Material 1: Table S1 Significant SNPs in different environments.

## Data Availability

All genome sequences have been uploaded to the National Center for Biotechnology Information under accession number PRJNA1203952 (https://www.ncbi.nlm.nih.gov/bioproject/PRJNA1203952).

## Abbreviations

MWS: Maize White Spot
RILs: Recombinant Inbred Lines
SNP: Single Nucleotide Polymorphism
QTL: Quantitative Trait Loci
GWAS: Genome-Wide Association Study
WGRS: Whole-Genome Resequencing
BLUP: Best Linear Unbiased Prediction
LD: Linkage Disequilibrium
PVE: Phenotypic Variation Explained
qRT-PCR: Quantitative Real-Time PCR
CDS: Coding Sequence
